# Teratogen Potential Evaluation of the Aqueous and Hydroalcoholic Leaf Extracts of *Crataegus oxyacantha* in Pregnancy Rats

**DOI:** 10.3390/plants12122388

**Published:** 2023-06-20

**Authors:** Fany Renata Aguilera-Rodríguez, Ana Lourdes Zamora-Perez, Rosalinda Gutiérrez-Hernández, Sol María Quirarte-Báez, Claudia Araceli Reyes Estrada, Yveth Marlene Ortiz-García, Blanca Patricia Lazalde-Ramos

**Affiliations:** 1 Maestría en Ciencias y Tecnología Química, Unidad Académica de Ciencias Químicas, Universidad Autónoma de Zacatecas, Zacatecas 98000, Mexico; fany333@hotmail.com; 2 Instituto de Investigación en Odontología, Centro Universitario de Ciencias de la Salud, Universidad de Guadalajara, Jalisco 44340, Mexico; anazamora@gmail.com (A.L.Z.-P.); ortizgamarlene@hotmail.com (Y.M.O.-G.); 3 Licenciatura en Nutrición, Unidad Académica de Enfermería, Universidad Autónoma de Zacatecas, Zacatecas 98000, Mexico; rosalinda@uaz.edu.mx; 4 Instituto Mexicano del Seguro Social, Zacatecas 98000, Mexico; sol.quirarte@imss.gob.mx; 5 Maestría en Ciencias de la Salud, Unidad Académica de Medicina Humana, Universidad Autónoma de Zacatecas, Zacatecas 98000, Mexico; c_reyes13@yahoo.com.mx

**Keywords:** *Crataegus oxyacantha*, teratogen potential, micronuclei, malondialdehyde, cyclophosphamide

## Abstract

*Crataegus oxyacantha* is used in the treatment of cardiovascular diseases. The aim of this study was to evaluate the transplacental genotoxicity effect of aqueous (AE) and hydroalcoholic extract (HE) of leaves *C. oxyacantha* in a rat model and the quantification of malondialdehyde (MDA) in the liver. Three different doses of the AE and HE of the *C. oxyacantha* leaf were administered orally (500, 1000 and 2000 mg/kg) to Wistar rats during 5 days through the pregnancy term (16–21 days), and sampling in rats occurred every 24 h during the last 6 days of gestation, while only one sample was taken in neonates at birth. A sample of the mother’s and the neonate’s liver was taken for the determination of MDA. The results show that, at the hepatic level, the evaluated doses of extracts *C. oxyacantha* in pregnant rats and their pups did not show cytotoxicity. However, the AE and HE generated cytotoxic and genotoxic damage in the short term. On the other hand, only the AE showed a teratogenic effect. Based on these results, the AE and HE of the *C. oxyacantha* leaf should not be administered during pregnancy.

## 1. Introduction

The World Health Organization (WHO) has reported that around 80% of the world’s population depends on the use of medicinal plants [1]. The study of plants for medicinal purposes consists of different steps in their preclinical stage, such as the selection of plants to be investigated, correct botanical identification, phytochemical characteristics, and pharmacological and toxicological studies [2].

Tests for the detection of agents that damage DNA are of great importance, since genotoxic compounds can alter the genetic material in organisms [3], which can manifest itself in teratogenic effects and germ cell mutations, influence aging processes [3,4], and induce somatic cell mutations that can lead to cancer development [4,5,6,7].

When the damage is generated in pregnancy, the compound is called a teratogen [8], since it can alter the genetic material, causing mutations in somatic and germ cells [9]. Various chemical agents can cause damage at birth, whether physiological or biochemical, at any stage of development of the fetus, causing either uterine death, abortion, premature birth, and neonatal poisoning [10].

The teratogenic potential is associated with the formation of the micronucleus [11,12]. Any compound that can cross the placental barrier and induce micronucleated erythrocytes in the fetus is considered a potential teratogen [10].

The micronucleus technique allows us to determine the ability of a compound to generate chromosomal damage (clastogenic or aneugenic) in the prenatal period, when the mother has been exposed to it, which would lead to a mutagenic risk [13,14].

Among the plants with medicinal purposes is *C. oxyacantha*, which is a shrub and a member of the Rosaceae family [15].

Used since ancient times mainly in treating cardiovascular diseases [16,17,18,19,20,21,22,23], its activity has been described as lipid-lowering [24,25,26], immunomodulatory [27,28], hepatoprotective [29,30,31], anti-inflammatory [32,33], antioxidant [32,33,34,35,36,37], antimicrobial [33,37,38], anxiolytic, and antidepressant [39,40]. These activities have been associated with the different types of flavonoids that are present in the leaf, bark, fruit, and flowers of *C. oxyacantha*. However, these also have a close relationship with the toxicological potential of the plant. According to the reported studies, the toxicological profile of *C. oxyacantha* has not yet been completely determined, as only the genotoxicity and cytotoxicity of the fruit have been described both in vivo and in vitro, as well as the average lethal dose of the leaf. Therefore, the present study aims to determine the teratogenic potential of the AE and HE of the leaves of *C. oxyacantha* in Wistar rats and their babies. 

## 2. Results

### 2.1. Phytochemical Analysis Leaf of C. oxyacantha

The presence of flavonoids, tannins, and quinines were identified by phytochemical analysis, and the results are shown in Table 1. 

The AE and HE leaf *C. oxyacantha* extract showed the presence of derivatives of gallic acid and catechol compounds with the γ-benzopyrone nucleus and anthraquinones. The HE showed the presence of anthrone derivatives.

### 2.2. Proportions of Polychromatic Erythrocytes (PCEs) and Micronucleated Polychromatic Erythrocytes (MNPCEs) in Pregnant Rats

The results of the proportions of PCEs and MNPCEs of the AE and HE leaf of *C. oxyacantha* in pregnant rats of the Wistar strain are shown in Table 2.

The number of PCEs and MNPCEs at the different sampling times did not present significant changes in its baseline value (0 h). However, the positive control (cyclophosphamide, CP) decreased the proportion of PCEs significantly at 120 h and statistically significantly increased the proportion of MNPCEs at 96 and 120 h with respect to its baseline value.

Similarly, the AE and HE leaf of *C. oxyacantha* decreased the PCEs in the three doses evaluated (Table 2). The 2000 mg/kg dose of the AE showed a significant decrease to its basal value from 24 to 120 h; likewise, the dose of 2000 mg/kg dose of the HE showed a decrease from 72 to 120 h. The 1000 mg/kg dose of the AE and HE decreased this proportion statistically significantly from 48 to 120 h. The 500 mg/kg dose of the HE showed a statistical decrease at 48, 96, and 120 h; however, the 500 mg/kg dose of the AE only showed a significant decrease in this proportion at 120 h. 

Regarding the proportion of MNPCEs, between the AE and HE of the *C. oxyacantha* leaf, only doses of 2000 mg/kg showed a significant increase to its basal value, with the AE at 72 and 96 h (*p*-value = 0.002 and 0.025, respectively), and the HE only at 72 h (*p*-value = 0.011).

### 2.3. Proportion of PCEs, MNPCEs and Micronucei (MNs) in Neonates of Rats

The teratogenic potential was evaluated in the peripheral blood of the neonates of rats exposed and not exposed to the AE and HE leaf of *C. oxyacantha* by the MN test. The results obtained are presented in Table 3.

Concerning the proportion of PCEs, only the neonates of the rats exposed to CP and the dose of 2000 mg/kg of the AE leaf of *C. oxyacantha* showed a statistically significant decrease compared to the negative control, with a *p-value* = 0.000 (Table 2).

The proportion of MNPCEs in the neonates of rats exposed to the leaf extracts of the *C. oxyacantha* showed a dose-dependent increase, which is more noticeable in the AE. The neonates of the rats exposed to CP (60 mg/kg) and the 2000 mg/kg dose of the AE leaf of *C. oxyacantha* showed a statistically significant increase compared to the negative control proportion, with a *p-value* = 0.000. In contrast, the neonates of rats exposed to the 500 mg/kg dose of HE leaf of *C. oxyacantha* showed a significant decrease in this proportion.

The proportion of MNEs obtained from neonates exposed to CP compared to neonates that were not exposed (negative control) showed a statistical increase in this proportion (*p*-value = 0.0001). The neonates of rats exposed to the AE leaf of *C. oxyacantha* showed results very similar to the three doses evaluated in the neonates of the non-exposed rats, for which no significant differences were found between them. In contrast, the neonates of rats exposed to the evaluated doses of HE leaf showed a statistically significant decrease in this proportion to the neonates of the not exposed rats (Table 2).

### 2.4. Hepatic Peroxidation

**Figure 1:**
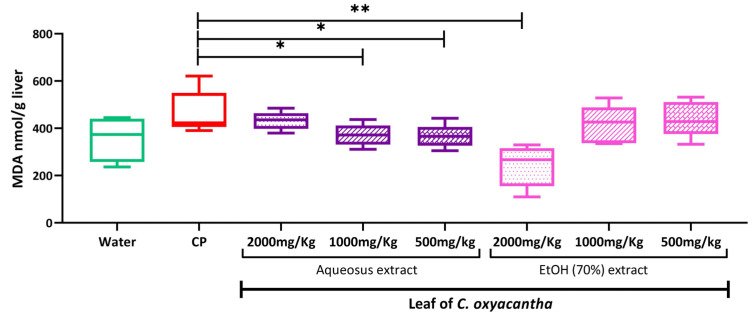
MDA concentration in the liver of Wistar rats exposed to aqueous and hydroalcoholic extracts of the *C. oxyacantha* leaf at the end of pregnancy. The results are expressed as the median with the minimum and maximum. Intergroup comparisons were made using the Kruskall–Wallis analysis with Dunn’s post hoc and were estimated to be statistically significant when * *p* < 0.05 and ** *p* < 0.001. MDA, malondialdehyde; nmol, nanomole; g, grams; kg, kilograms.

Figure 1 shows the results obtained for the concentration of MDA at the liver level in rats at the term of gestation. The group treated with CP showed the highest concentration of MDA in the liver compared to the other groups evaluated.

The AE leaf of *C. oxyacantha* tends to increase the concentration of MDA as the dose increases, in contrast, the groups treated with the HE of *C. oxyacantha* show a tendency to decrease MDA as the dose increases, not being statistically significant for these differences.

When comparing the three evaluated doses of the AE and HE to the CP group, the medium and low dose of the AE and the high dose of the HE leaf of the *C. oxyacantha* showed a statistically lower concentration of MDA compared to the CP group (*p*-value = 0.047, 0.047, and 0.009, respectively).

**Figure 2:**
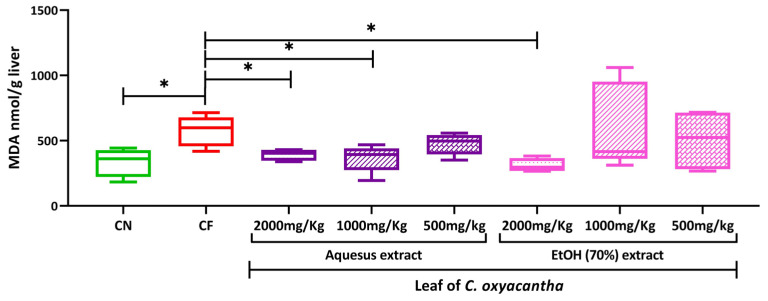
MDA concentration at a hepatic level in neonates of Wistar rats exposed to different doses of aqueous and hydroalcoholic leaf extracts of *C. oxyacantha*. The results are expressed as the median with the minimum and maximum. Intergroup comparisons were made using the Kruskall–Wallis analysis with Dunn’s post-hoc and were estimated to be statistically significant when * *p* < 0.05. MDA, malondialdehyde; nmol, nanomole; g, grams; kg, kilograms.

Figure 2 shows the MDA concentration in the liver of neonates of rats exposed to the different doses of the AE and HE leaf of *C. oxyacantha*.

The neonates of mothers exposed to CP presented a higher concentration than that presented by the neonates of mothers exposed only to water, this difference being statistically significant (*p*-value = 0.032). The MDA concentrations of the neonates of the rats exposed to the 2000 and 1000 mg/kg doses of the AE were statistically lower than those of the neonates of mothers exposed to CP (*p*-value = 0.028 and 0.027, respectively). Similarly, the neonates of rats exposed to the 2000 mg/kg dose of the HE also presented MDA concentrations lower than those of the neonates of rats exposed to CP (*p*-value = 0.0014).

## 3. Discussion

According to the WHO, approximately 80% of the world population resorts to the use of medicinal plants; however, there is a great gap in the knowledge of the chemical compositions, mechanisms of action, as well as the safety and efficacy of these plants [41,42,43].

Pregnancy is a condition that should be considered a time of minimal medical intervention, even in the consumption of plant-based products. This is because it has been described that a wide variety of congenital deformities usually occur in the fetus during the period of organogenesis [44]. Mainly, it is because xenobiotic-metabolizing enzymes are induced during pregnancy, which can increase the metabolism of secondary metabolites that are substrates of these enzymes, causing intoxication by them [45].

The MN test in peripheral blood allows the cytotoxicity and genotoxicity of an agent to be evaluated, based on the decrease in the number of PCEs and the increase in MNPCE in peripheral blood [46].

It has been described that the presence of MN in the peripheral blood of neonates can assess the teratogenic potential of xenobiotics administered during pregnancy, since it has been shown that many genotoxic compounds have teratogenic potential and, in turn, could involve various mechanisms of teratogenicity [47,48]. MN is easily observable in erythrocytes obtained from newborn rats, due to the immaturity and hypofunctionality of the neonatal spleen [49].

The teratogenic potential was evaluated in newborn rats, which were exposed to the different evaluated doses of the AE and HE of the *C. oxyacantha* leaf at the end of the organogenesis period (from day 16 to day 21 of the gestation period).

As a positive control, CP was achieved, which is activated by cytochrome P-450 enzyme to mustard phosphoramide and acrolein. The group exposed to CP increased the proportion of PCE and increased the number of MNPCE in a statistically significant way, both in pregnant rats and in their neonates. These results confirm its cytotoxic and genotoxic effects, since acrolein has been described as the metabolite with the highest cytotoxic activity of CP, generating mitochondrial dysfunction, endoplasmic reticulum stress, and activation of apoptotic transcripts [50,51].

In a previous study, it was reported that the doses of 2000 and 1000 mg/kg of the AE and HE of *C. oxyacantha* in 12-week-old Balb-c mice had no effect on the proportion of EPCS [52]. However, in this study, it was observed that pregnant rats exposed to doses of 500, 1000, and 2000 mg/kg of the AE and HE of the *C. oxyacantha* leaf statically decreased the proportion of PCE to the basal value. The fact that, in pregnant rats, at the lowest dose evaluated, which was 500 mg/kg of the AE and HE of the *C. oxyacantha* leaf, a cytotoxic effect was observed, may be because, during pregnancy, the xenobiotic-metabolizing of some enzymes is induced, which can increase the metabolism of secondary metabolites, namely the substrates of these enzymes [45].

The group treated with CP presented a teratogenic effect by significantly decreasing the proportion of PCEs and significantly increasing the number of MNPCEs and MNEs in the peripheral blood of rat neonates. Previously, the transplacental effect of CP has been reported, which has been visualized with induction of MN in rat neonatal peripheral blood erythrocytes, fetal liver cells, and rat and mouse amniotic fluid cells exposed to CP during gestation [53,54,55,56]. 

Its teratogenic effect is associated with phosphoramide mustard and acrolein, the active forms of CP, which are obtained through the metabolism of microsomal monooxygenases of cytochrome P-450. Mainly, they have an alkylating effect on DNA, RNA, and embryonic proteins [57].

The AE of the leaf of *C. oxyacantha* in rat neonates exposed to a 2000 mg/kg dose showed a cytotoxic and genotoxic effect by decreasing the proportion of PCEs and increasing that of MNPCEs. In contrast, neonates of rats exposed to different doses of the HE did not show cytotoxic or genotoxic damage. A previous study showed genotoxic and cytotoxic damage to the leaf and bark of *C. oxyacantha* in 12-week-old Balb-c mice (2000 mg/kg) by showing significant changes in the proportion of MNPCEs [52].

However, so far, no reports have been found evaluating the teratogenic potential of *C. oxyacantha* to compare with our results.

There are reports of the antioxidant effect of flavonoids, which have been related to other types of pharmacological activity, such as anti-inflammatory effects, and its protective effect on the liver, brain, and cardiovascular levels. However, it has also been described that they have pro-oxidant effects, which lead to DNA damage and the formation of MN, chromosomal aberrations, and mutations. These effects are closely related to the experimental conditions under which the compounds are evaluated. Furthermore, Schröder-van der Elst et al. showed that flavonoids can cross the placenta in rats and accumulate in fetal tissues [58].

The difference in genotoxic and cytotoxic effects between the AE and HE of the leaf of *C. oxyacantha* may be due to the concentration of secondary metabolites, which varies according to the type of solvent used. Both extracts showed flavonoids, tannins, and quinones; however, we do not know in what proportion they were found, nor which one specifically contained which metabolites. 

Benabderrahmane et al., in 2018, determined some polyphenols present in the leaves of *C. oxyacantha*, such as caftaric acid, caffeic acid, chlorogenic acid, orientin, miquelianin, rutin, and apigenin [59]. 

Other authors have also reported the presence of epicatechin (dimer B2, B4, B5; trimer C1; tetramer D1; pentamer E1), isoquercitrin, hiperoside, isovitexin, and vitexin in the leaf of *C. oxyacantha* [60,61,62]. Apigenin, one of the compounds present in the leaves of *C. oxyacantha*, has been described as having a slow metabolism, which allows its accumulation in the body [58]; there are also studies that demonstrate that it generates a teratogenic potential in rat embryos by causing a decrease in the weight, as well as in the size of the skull and tail [63]. This can be related to the antiestrogenic effect of apigenin, which makes it difficult for the gestation process to be carried out correctly [64].

On the other hand, some epicatechin derivatives, which are also found in the leaves of *C. oxyacantha* at low concentrations, activate signaling pathways that regulate homeostasis. However, when concentrations increase, other pathways are activated, such as caspases that lead to a cytotoxic effect mediated by apoptosis [64]. Likewise, it has been described that the metabolism of flavonoids forms phenoxyl radicals which cause toxicity in the mitochondria, leading the cell to a state of apoptosis [65]. It has been shown that the methanolic extract of *C. oxyacantha* fruit has genotoxic effects in cultured human lymphocytes and generates mutations in bacteria of the Salmonella typhimurium strain [66].

When determining the concentration of MDA in the liver, the group treated with CP showed the highest concentration of MDA in the liver. It has been described that the secondary metabolites of CF, such as phosphoramide mustard and acrolein, have pro-oxidant activity, which is related to its toxicity. Acrolein has a short half-life; however, it is considered the metabolite that unchains a higher production of reactive oxygen species, which causes lipid peroxidation and oxidative DNA damage [67,68]. Similarly, it has been reported that approximately 10% of CF is metabolized to reactive aldehydes, such as chloroacetaldehyde and dichloroethylcyclophosphamide, which also generates a pro-oxidant effect. CF exposure during gestational organogenesis has also been reported to cause a variety of fetal abnormalities in mice, rats, rabbits, and humans [69]. El-Dakdoky (2015) showed that CF administered intraperitoneally at a dose of 12 mg/kg in rats on the 13th day of gestation caused damage to the products by showing an increase in the concentration of MDA in the fetal liver [70]. 

The present study shows that the evaluated doses of the AE and HE of *C. oxyacantha* in pregnant rats and their neonates did not show hepatic cytotoxicity. 

There are few studies on the evaluation of the safety of medicinal plants during pregnancy, for which no reports were found in which the quantification of MDA at the liver level in pregnant and neonatal rats exposed to these extracts has been evaluated.

It has been described that the fruit of *C. oxyacantha* at doses of 200 mg/kg administered orally for 7 days in mice generates cytotoxicity at the liver level (hepatocytes with more acidophilic cytoplasm, formation of vacuoles and space in intercellular cells, increased lumen of sinusoidal capillaries, and increased hepatic tissue defense cells) [71]. 

However, the cytoprotective effect of the hydroalcoholic extract (EtOH) of the fruit of *C. oxyacantha* has also been described by decreasing the concentration of MDA in rats exposed to doses of 10 and 20 mg/kg for ten days and a dose of 50 mg/kg for twelve weeks [29]. 

Moreover, it was described that the n-butanol extract of *C. oxyacantha* leaves at a dose of 100 mg/kg in rats decreased MDA concentrations in the liver [31]. Vanhees and collaborators investigated the effects of maternal quercetin exposure in mice. They showed that during embryonic development, exposure increased iron levels and significantly decreased oxidative stress at the liver level [72].

Although the antioxidant effect of flavonoids, which are the main chemical compounds present in *C. oxyacantha*, is known, some studies show that they have a dual effect, such as the case of apigenin, which is a flavone present in *C. oxyacantha* leaf; this compound has a pro-oxidant effect when administered alone in murine models [58,66].

Quercetin is another of the metabolites present in *C. oxyacantha*, and this is one of the most abundant flavonols and is distributed in different foods. Various studies have shown that its consumption is safe during pregnancy, in addition to helping to reduce the concentration of MDA at the cardiac level and increasing the activity of antioxidant enzymes in embryos of rats treated with theophylline [73]. Another study reveals that rutin (a flavonol glycoside composed of quercetin) administered during gestation and lactation to female C57BL/6J mice modifies the concentrations of minerals, such as calcium, at the hepatic level in their offspring [74].

## 4. Materials and Methods

### 4.1. Materials and Reagents

The reagents employed were of the commercial brand J. T Baker (Mexico) and Golden Bell (Mexico). Cyclophosphamide (CAS 6055 19-2) and acridine orange (CAS 10127-02-3) were from Sigma-Aldrich (St. Louis, MO, USA).

### 4.2. Plant Material

The leaf of *C. oxyacantha* was obtained from the supplier Nutra Herbal de Mexico (Convento de Balvanera #24, Col. Jardines de Santa Monica, Mexico, Tlalnepantla C.P. 54050, Mexico).

### 4.3. Preparation of the Aqueous and Hydroalcoholic Leaf Extracts of C. oxyacantha

The dried leaves of *C. oxyacantha* were pulverized. A decoction was made to obtain the AE, with a ratio of 1 g per 10 mL of water, boiled for 15 min, then filtered and lyophilized.

For the HE of *C. oxyacantha*, 70% ethanol was used, and this was carried out by mechanical maceration for 48 h. The solution was refluxed for 2 h and filtered. Activated carbon was added to remove chlorophyll and the ethanol was removed with a rotary evaporator. Finally, it was lyophilized.

### 4.4. Phytochemical Analysis Leaf of C. oxyacantha

The phytochemical screening evaluation was performed through colorimetric tests to detect the presence or absence of phytochemical constituents (flavonoids, tannins, and quinines).

Phytochemical screening of the extracts was performed using the following reagents and chemicals: flavonoids with the sodium hydroxide reagent test and Shinoda test and Z; tannins with the gelatin test, ferric chloride reagent test, and potassium ferrocyanide reagent test. They were identified by characteristic color changes and precipitation reactions using standard procedures [75].

### 4.5. Animals

Forty clinically healthy 3-month-old pregnant Wistar rats were placed in polycarbonate cages with food and water (Harlan Teklad Lab Block) ad libitum. The animals were provided by the Claude Bernard Biotherium of the Health Sciences Area, Campus UAZ, Siglo XXI, of the Autonomous University of Zacatecas.

### 4.6. Study Groups 

The teratogenic potential was evaluated in the neonates of 40 female rats of the Wistar strain between 2–3 months of age, with an average weight of 205.10 g ± 10.75 g, as well as the genotoxic and cytotoxic damage of the AE and HE of *C. oxyacantha* in mothers. The animals were divided into 8 experimental groups: Group 1 received sterile water (negative control); Group 2 received 60 mg/kg of cyclophosphamide (CP) divided into two doses (positive control); Group 3, high dose, received 2000 mg/kg of the AE; Group 4, medium dose, received 1000 mg/kg of the AE; Group 5, low dose, received 500 mg/kg of the AE; Group 6 also received a high dose, 2000 mg/kg of the HE; Group 7, medium dose, received 1000 mg/kg of the HE; Group 8, low dose, received 500 mg/kg of the HE. The administration of the extracts was carried out orally through the esophageal cannula for 5 days, with a volume 0.1 mL/10 g of weight.

### 4.7. Mating

**Figure 3:**
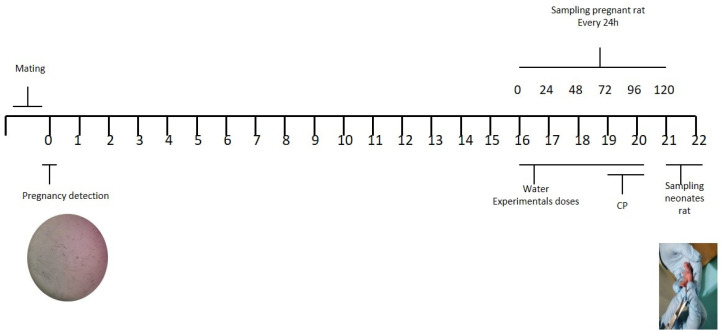
Scheme for the evaluation of genotoxicity of the aqueous and hydroalcoholic leaf extracts of *C. oxyacantha* in rats and their neonates (taken and modified from Morales-Velazquez, G., et al., 2019) [56].

The rats were mated with the male for one week. Pregnancy was confirmed by a vaginal flush with 0.1 mL of sterile water using a micropipette. The flush was placed on a slide, which was observed by 10× optical microscopy to detect the presence of sperm, which indicated the onset of gestation (day zero). In addition, the visualization of the vaginal plug confirmed pregnancy. Once the pregnancy of the female was confirmed, the gestation period was scheduled and the administration of the corresponding dose was scheduled in the last days of pregnancy (days 16 to 21), as shown in Figure 3.

### 4.8. Sample Preparation and Micronucleus Analysis in Pregnancy Rats and Their Neonates

The evaluation of cytotoxic and genotoxic damage in pregnant rats was determined by the micronucleus test (MN) [76]. Peripheral blood smears of the rats were made at 0, 24, 48, 72, 96, and 120 h after the administration of the different doses, for which a drop of blood was obtained from the tip of the tail of the animals, of each group.

Once their gestation time was completed, 6 neonates were selected per rat, a blood sample was taken from the tail of each neonate, and a duplicate spread was made. The smears were fixed in ethanol for 10 min and stained with acridine orange. An Olympus CX31 microscope equipped with epifluorescence and an oil immersion objective (100×) was used to evaluate the genotoxic and cytotoxic damage. The number of polychromatic erythrocytes (PCEs) was counted in 1000 total erythrocytes (TEs), the number of micronucleated polychromatic erythrocytes (MNPCEs) in 1000 PCEs, and the number of micronucleated erythrocytes (MNEs) in 10,000 TEs [77].

### 4.9. Hepatic Peroxidation (Malondialdehyde Quantification, MDA)

The quantification of MDA in the liver was carried out by the modified method of Mihara and Uchiyama in 1978. A 10% liver homogenate was prepared with 1.15% KCl; 0.05 mL of the homogenate was taken and added to a tube, 3 mL of 1% H3PO4 and 0.3 mL of 0.6% of TBA were added, the mixture was put in a water bath for 45 min, cooled, and then 1-butanol was added. The MDA concentration was determined using a spectrophotometer at a wavelength of 534 nm [78].

### 4.10. Statistical Analysis

For the frequencies of PCEs, MNPCEs, and MNEs, the results obtained were expressed as mean ± standard deviation per group. For rats, comparisons were made between each group and its respective baseline value (0 h), using the analysis of variance (ANOVA) for repeated measures and the Bonferroni adjustment test for multiple post hoc comparisons. In the case of neonates, intergroup comparisons were made concerning negative control values, using one-way analysis of variance (ANOVA), and the Dunnett adjustment test was used for multiple post hoc comparisons. 

Data for MDA concentrations were expressed as a median with the maximum and minimum. Intergroup comparisons were made using the Kruskall–Wallis analysis with Dunn’s post hoc.

Statistical significance was set at *p* < 0.05. Data analysis was performed using the IBM SPSS (V25) statistics program for Windows.

### 4.11. Ethical Considerations

The handling of the animals was based on the Official Mexican Standard NOM-062-ZOO-1999, which shows the specifications and techniques for the production, care, and use of institutional laboratory animals. The sacrifice was based on the NOM-033-SAG/ZOO-2014 and the NOM-087-ECOL-SSA1-2002. The project has the endorsement of the Bioethics Committee of the Health Sciences Area of the Autonomous University of Zacatecas with the number ACS/UAZ/051/2019.

## 5. Conclusions

The AE and HE leaf of *C. oxyacantha* showed cytotoxic effect at the three doses evaluated and genotoxic effects at the doses of 2000 mg/kg in pregnant Wistar rats. Similarly, the 2000 mg/kg dose of the AE of the leaf of *C. oxyacantha* was shown to have a teratogenic potential. The pregnant rats and their neonates exposed to the AE and HE leaf of *C. oxyacantha* did not show hepatic cytotoxicity. Based on the results obtained in this model, it is recommended not to administer AE and HE of *C. oxyacantha* leaves to pregnant women. The importance of these findings is to contribute to the safety profile of *C. oxyacantha* leaf extracts during pregnancy for both the mother and the fetus.

## Figures and Tables

**Table 1:** Results of the phytochemical analysis of leaf of *C. oxyacantha*.

Test		Leaf	
		Hydroalcoholic Extract	Aqueous Extract
Flavonoids	Shinoda HCl_(c)_	−	−
	NaOH	+	+
Tannins	Gelatin	−	−
	FeCl_3_	+	+
	Potassium ferrocyanide	−	−
Quinines	NH_4_OH	+	+
	H_2_SO_4_	−	−
	Bornträger reaction	+	−

+: present; −: absent; _(c)_: concentrate

**Table 2:** Number of PCEs and MNPCEs at different sampling times in the study groups in pregnant rats.

PCEs/1000 TEs								
			0 h	24 h	48 h	72 h	96 h	120 h
Controls		SW	30.40 ± 4.33	29.80 ± 5.16	28.00 ± 3. 53	30.40 ± 3.57	30.20 ± 2.58	28.60 ± 2.70
		*p-value*	-----	1.00	1.00	1.00	1.00	1.00
		CP	53.00 ± 5.35	52.00 ± 5.35	55.00 ± 9.27	52.00 ± 12.30	46.75 ± 14.50	12.00 ± 4.54
		*p-value*	------	1.00	0.610	1.00	0.346	0.0001
*C. oxyacantha*	Aqueous Ext of leaf	2000 mg/kg	39.40 ± 2.19	31.20 ± 2.38	28.00 ± 2.91	25.00 ± 2.34	22.60 ± 3.28	22.40 ± 3.50
		*p-value*	------	0.002	0.0001	0.0001	0.0001	0.0001
		1000 mg/kg	40.20 ± 4.38	34.40 ± 3.64	31.00 ± 1.58	29.00 ± 2.54	32.40 ± 4.03	29.40 ± 4.92
		*p-value*	------	0.065	0.0001	0.0001	0.033	0.002
		500 mg/kg	38.00 ± 4.41	40.20 ± 4.91	33.60 ± 2.96	34.40 ± 2.79	35.00 ± 2.34	27.80 ± 3.03
		*p-value*	------	1.00	0.172	1.000	1.000	0.004
	Hydroalcoholic Ext of leaf	2000 mg/kg	39.80 ± 3.83	38.00 ± 2.91	36.00 ± 2.12	32.60 ± 3.04	28.80 ± 1.92	24.80 ± 1.64
		*p-value*	------	1.00	0.405	0.025	0.001	0.0001
		1000 mg/kg	41.40 ± 2.60	37.20 ± 2.16	34.20 ± 1.09	31.00 ± 1.58	27.20 ± 3.49	24.60 ± 2.50
		*p-value*	-------	0.498	0.002	0.0001	0.0001	0.0001
		500 mg/kg	49.60 ± 2.07	44.00 ± 2.91	44.20 ± 5.63	44.40 ± 2.96	40.20 ± 1.09	39.20 ± 2.48
		*p-value*	------	0.085	0.037	0.278	0.005	0.003
**MNPCEs /1000 PCEs**								
Controls		SW	3.20 ± 1.48	4.00 ± 1.00	3.40 ± 1.51	3.00 ± 1.22	2.80 ± 1.09	4.20 ± 0.44
		*p-value*	-----	1.00	1.00	1.00	1.00	1.00
		CP	5.00 ± 0.81	5.50 ± 1.73	6.75 ± 1.70	6.000 ± 2.58	10.75 ± 2.21	18.50 ± 4.35
		*p-value*	----	1.00	0.446	1.00	0.0001	0.0001
*C. oxyacantha*	Aqueous Ext. of leaf	2000 mg/kg	2.60 ± 0.89	3.40 ± 0.89	4.40 ± 1.67	5.20 ± 1.48	5.00 ± 2.12	3.60 ± 1.34
		*p-value*	-----	1.00	0.202	0.002	0.025	1.00
		1000 mg/kg	3.00 ± 1.22	4.20 ± 1.64	4.80 ± 1.92	3.60 ± 0.54	5.00 ± 2.34	4.20 ± 2.16
		*p-value*	-----	0.567	0.202	1.00	0.111	1.00
		500 mg/kg	2.80 ± 1.09	3.00 ± 0.70	3.80 ± 1.30	4.40 ± 0.89	2.40 ± 1.14	1.40 ± 0.54
		*p-value*	-----	1.00	1.00	0.155	1.00	1.00
	Hydroalcoholic Ext of leaf	2000 mg/kg	2.40 ± 0.54	2.20 ± 1.30	3.00 ± 1.00	4.60 ± 0.89	3.60 ± 0.54	3.00 ± 1.22
		*p-value*	-----	1.00	1.00	0.011	1.00	1.00
		1000 mg/kg	4.00 ± 0.70	3.60 ± 1.34	2..80 ± 0.83	3.20 ± 1.30	2.40 ± 0.89	2.80 ± 1.30
		*p-value*	----	1.00	1.00	1.00	0.433	1.00
		500 mg/kg	2.60 ± 1.14	2.20 ± 0.44	2.60 ± 0.89	3.20 ± 1.30	4.00 ± 1.22	3.20 ± 1.30
		*p-value*	-----	1.00	1.00	1.00	0.806	1.00

The results are expressed as mean ± standard deviation. Comparisons were made between each group and their respective baseline value (0 h), using the analysis of variance (ANOVA) for repeated means and the Bonferroni adjustment test was used for multiple post hoc comparisons. Results were considered statistically significant when *p* < 0.05. Abbreviations are as follows SW, sterile water; CP, cyclophosphamide; Ext, extract; PCEs, polychromatic erythrocytes; TEs, total erythrocytes; MNPCEs, micronucleated polychromatic erythrocytes; h, hour.

**Table 3:** Proportion of PCEs, MNPCEs, and MNEs in peripheral blood of rat neonates exposed and not exposed to the aqueous and hydroalcoholic leaf extracts of *C. oxyacantha*.

			Number of Newborns	PCEs/1000 TEs	MNPCEs/1000 PCEs	MNEs/10,000 TEs
Controls		Negative control (SW)	30	678.56 ± 72.57	4.87 ± 1.35	7.00 ± 2.01
		Positive control (CP)	30	500 ± 93.23	27.07 ± 10.63	13.33 ± 5.33
		*p-value*		**0.000**	**0.000**	**0.000**
Aqueous Ext of leaf		2000 mg/kg	30	532.66 ± 84.48	8.30 ± 1.98	8.17 ± 1.70
		*p-value*		**0.000**	**0.000**	0.384
		1000 mg/kg	30	618.70 ± 78.05	6.03 ± 1.92	6.73 ± 1.63
		*p-value*		0.083	0.208	1.000
		500 mg/kg	30	658.30 ± 95.59	5.50 ± 1.30	7.43 ± 1.87
		*p-value*		1.00	0.835	1.000
Hydroalcoholic Ext. of leaf		2000 mg/kg	30	636.70 ± 98.84	4.40 ± 1.07	5.40 ± 1.65
		*p-value*		0.813	0.977	**0.038**
		1000 mg/kg	30	652.73 ± 80.84	4.50 ± 1.35	5.27 ± 1.66
		*p-value*		0.995	1.000	**0.017**
		500 mg/kg	30	661.06 ± 107.06	3.23 ± 0.93	4.23 ± 1.38
		*p-value*		1.00	**0.000**	**0.000**

The results are expressed as mean ± standard deviation. Intergroup comparisons were made with respect to the negative control values, by means of the one-way analysis of variance (ANOVA), and the Dunnett’s adjustment test was used for multiple post hoc comparisons; results were considered statistically significant when *p* < 0.05 and were evidenced in boldface. Abbreviations are as follows: Ext, extract; PCEs, polychromatic erythrocytes; TEs, total erythrocytes; MNPCEs, micronucleated polychromatic erythrocytes; MNEs, micronucleated erythrocytes; SW, sterile water; CP, cyclophosphamide.

## Data Availability

The data presented in this study is available in the article.
